# Early predictors of severe left main and/or three‐vessel disease in patients with non‐ST‐segment elevation myocardial infarction: A dual‐center retrospective study

**DOI:** 10.1111/anec.13120

**Published:** 2024-05-05

**Authors:** Bihan Huang, Xueying Han, Yulian Huang, Dongdong Chen, Peiyi Xie, Shaoyuan Chen

**Affiliations:** ^1^ Department of Cardiology Huazhong University of Science and Technology Union Shenzhen Hospital Shenzhen China; ^2^ Department of Intensive Care Huazhong University of Science and Technology Union Shenzhen Hospital Shenzhen China; ^3^ Department of Cardiology The First Affiliated Hospital of Jinan University Guangzhou China; ^4^ Department of Cardiology The 6th Affiliated Hospital of Shenzhen University Medical School Shenzhen China

**Keywords:** aVR, left main and/or three‐vessel disease (LM/3VD), non‐ST‐segment elevation myocardial infarction (NSTEMI), predictor, SYNTAX score (SS)

## Abstract

**Background:**

Early detection of patients concomitant with left main and/or three‐vessel disease (LM/3VD) and high SYNTAX score (SS) is crucial for determining the most effective revascularization options regarding the use of antiplatelet medications and prognosis risk stratification. However, there is a lack of study for predictors of LM/3VD with SS in patients with non‐ST‐segment elevation myocardial infarction (NSTEMI). We aimed to identify potential factors that could predict LM/3VD with high SS (SS > 22) in patients with NSTEMI.

**Methods:**

This dual‐center retrospective study included a total of 481 patients diagnosed with NSTEMI who performed coronary angiography procedures. Clinical factors on admission were collected. The patients were divided into non‐LM/3VD, Nonsevere LM/3VD (SS ≤ 22), and Severe LM/3VD (SS > 22) groups. To identify independent predictors, Univariate and logistic regression analyses were conducted on the clinical parameters.

**Results:**

A total of 481 patients were included, with an average age of 60.9 years and 75.9% being male. Among these patients, 108 individuals had severe LM/3VD. Based on the findings of a multivariate logistic regression analysis, the extent of ST‐segment elevation observed in lead aVR (OR: 7.431, 95% CI: 3.862–14.301, *p* < .001) and age (OR: 1.050, 95% CI: 1.029–1.071, *p* < .001) were identified as independent predictors of severe LM/3VD.

**Conclusion:**

This study indicated that the age of patients and the extent of ST‐segment elevation observed in lead aVR on initial electrocardiogram were the independent predictive factors of LM/3VD with high SS in patients with NSTEMI.

## Graphical abstract

The degree of ST‐segment elevation observed in lead aVR and the age of patients were the independent predictive factors of LM/3VD with high SS in patients with non‐ST‐segment elevation myocardial infarction.

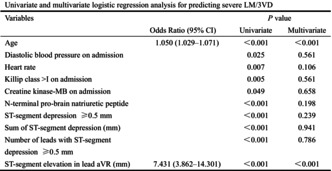

## 1 INTRODUCTION

Patients diagnosed with left main and/or three‐vessel disease (LM/3VD) are at an increased risk of poor prognosis (Zembala, 2019). The SYNTAX score (SS) was created as a means of quantifying the intricacy of coronary artery disease (CAD) and subsequently deciding the optimal revascularization approach for patients with LM/3VD. Patients with LM/3VD who have a high SS are more likely to be recommended for coronary artery bypass graft surgery (CABG). Early detection of patients concomitant with LM/3VD and high SS is crucial for determining the most effective revascularization options regarding the use of antiplatelet medications and prognosis risk stratification (Zembala, 2019). Hence, the identification of early and non‐invasive predictors to evaluate the severity of CAD would be clinically relevant. There are many studies that have been conducted to investigate the factors that can predict LM/3VD in patients with no–ST‐segment elevation acute coronary syndrome (NSTE‐ACS). Previous studies indicated that ST‐segment elevation in lead aVR was an independent predictor for LM/3VD and the extent of LM/3VD in comparison to ST‐segment deviation in other leads (D'Ascenzo et al., 2012; Kazemi et al., 2022). However, these studies did not evaluate LM/3VD severity by SS in patients with non‐ST‐segment elevation myocardial infarction (NSTEMI). In this research investigation, we conducted an assessment of the ability of the clinical factors on admission to predict LM/3VD with high SS in patients with NSTEMI. In this research, we evaluated the ability of clinical variables on admission to accurately predict LM/3VD with high SS in patients diagnosed with NSTEMI.

## 2 METHODS

### 2.1 Study population

This study retrospectively included a total of 481 consecutive patients diagnosed with acute NSTEMI at the Huazhong University of Science and Technology Union Shenzhen Hospital and the First Affiliated Hospital of Jinan University were retrospectively included in this study. Inclusion criteria were as follows: (1) first diagnosed with NSTEMI; (2) patients who performed coronary angiography procedures and had complete and evaluable angiography results. Patients with the following conditions were included in this research. (3) Typical ischemic chest pain occurred within 24 h before admission. The exclusion criteria employed in this research were as follows: (1) patients with previous CABG; (2) patients with takotsubo cardiomyopathy, myocarditis, chronic heart failure (heart failure with preserved ejection fraction, heart failure with mid‐range ejection fraction, or heart failure with reduced ejection fraction), cardiomyopathy, or pulmonary embolism; (3) patients who exhibit bundle branch block; and (4) patients with incomplete or inadequate data for analysis. The ethics committee of the Huazhong University of Science and Technology Union Shenzhen Hospital and the First Affiliated Hospital of Jinan University approved this study protocol.

### 2.2 Clinical data acquisition

Upon admission, relevant clinical variables were collected: (1) On admission, basic information such as age, gender, body mass index (BMI), cigarette use, heart beats per minute, systolic blood pressure, and diastolic blood pressure, as well as past medical history (diabetes, hyperlipidemia, and hypertension) were collected; (2) The initial blood sample results were analyzed, which encompassed the assessment of various parameters such as plasma glucose, serum creatinine, creatine kinase MB, cardiac troponin I, and N‐terminal pro‐brain natriuretic peptide (NT‐pro‐BNP); (3) The variables associated with coronary angiography, such as the SS, were collected; and (6) parameters of electrocardiogram (ECG).

### 2.3 Definitions

The diagnosis of acute NSTEMI was established through the symptom of ischemic chest discomfort, the detection of abnormal cardiac biomarkers, and/or the identification of new ischemic alterations in the ECG and was further reconfirmed by angiography (Thygesen et al., 2018).

An online calculation tool (http://syntaxscore.com/) was used to calculate the SS based on coronary angiography. This SS was calculated by two experienced cardiologists who were unaware of the baseline clinical parameters of patients. In case of any disagreement, discuss with the third author to reach a consensus. An SS greater than 22 was classified as a high SS. LM/3VD was defined as the presence of a stenosis equal to or greater than 50% in the diameter of the LM, and/or a stenosis equal to or greater than 50% in three major epicardial vessels. LM/3VD with SS ≤22 were classified as nonsevere LM/3VD. LM/3VD with an SS greater than 22 were classified as Severe LM/3VD.

Upon admission, 12‐lead ECGs were obtained using a paper speed of 25 mm/s and a magnification of 10 mm/mV. All ECGs were analyzed by experienced cardiologists who were unaware of the study protocol and any other clinical data of patients. The measurement of ST‐segment deviation was conducted at the J point (Wagner et al., 2009).

### 2.4 Statistical analysis

The normality of the distribution was evaluated using the Kolmogorov–Smirnov test. In statistical analysis, continuous parameters that follow a normal distribution are described using mean and standard deviation. Continuous parameters that do not adhere to a normal distribution are described using medians and interquartile range. In order to compare the measurement data between groups, statistical tests were employed, including the *t*‐test, one‐way analysis of variance, Mann–Whitney *U*‐test, or Kruskal–Wallis *H*‐test. These tests were chosen based on the assumption of normality. Categorical variables are described using percentages. In order to compare categorical variables, chi‐square analysis or Fisher's exact test were employed. Variables that had a *p*‐value lower than .05 in the univariate analyses were subsequently incorporated into logistic regression analysis to ascertain the independent predictive factors. For all tests, the level of significance was set at a *p*‐value of less than .05. The study employed receiver operating characteristic (ROC) curve analysis to identify the most suitable threshold, and the area under the curve (AUC) was utilized to assess the predictive capacity of the independent predictors. The statistical analyses were conducted utilizing SPSS version 26.0.

## 3 RESULTS

### 3.1 Univariate and multivariate logistic regression analysis results

Overall, 481 patients diagnosed with NSTEMI were included in this research. The average age of the included patients was 60.9 years, with 75.9% of them being male. The patients were categorized into three groups according to the severity of coronary artery disease, determined by SS. 244 (50.7%) patients in the without LM/3VD (non‐LM/3VD) group, 129 (26.8%) patients in the LM/3VD with SS ≤22 (nonsevere LM/3VD) group, and 108 (22.5%) patients in the LM/3VD with an SS greater than 22 (severe LM/3VD) group (Table 1).

**TABLE 1:** Clinical characteristics of the study population.

Variables	Non‐LM/3VD (*n* = 224)	LM/3VD		*p* Value
		Nonsevere (*n* = 129)	Severe (*n* = 108)	
Basic information				
Age (years)	58 [50, 68]	61 [51, 70]	68 [58, 77]	<.001
Men (%)	188 (77.0)	101 (78.3)	76 (70.4)	.304
Obesity body mass index ≥30 kgm^2^	42 (17.2)	28 (21.7)	15 (13.9)	.281
Hypertension (%)	138 (56.6)	77 (59.7)	71 (65.7)	.269
Diabetes mellitus (%)	80 (32.8)	57 (44.2)	48 (44.4)	.034
Hyperlipidemia (%)	127 (52.0)	76 (58.9)	51 (47.2)	.188
Smoking (%)	106 (43.4)	64 (49.6)	42 (38.9)	.244
Family history of coronary artery disease (%)	8 (3.3)	6 (4.7)	3 (2.8)	.764
Symptom onset ≤6 h (%)	77 (31.6)	27 (20.9)	33 (30.6)	.083
Systolic blood pressure on admission (mmHg)	135 [121, 150]	134 [118, 152]	134 [121, 150]	.636
Diastolic blood pressure on admission (mmHg)	84 [73, 91]	80 [72, 88]	77 [70, 88]	.008
Heart rate (bpm)	78 [68, 86]	78 [66, 86]	82 [70, 94]	.025
Killip class >I on admission (%)	33 (13.5)	29 (22.5)	31 (28.7)	.002
Laboratory results				
Plasma glucose (mmol/L)	5.9 [5.3, 7.0]	6.3 [5.3, 8.1]	6.1 [5.3, 9.0]	.109
Serum creatinine (μmol/L)	75.6 [63.0, 87.7]	77.3 [62.4, 95.3]	76.7 [65.9, 97.8]	.282
Cardiac troponin I (ng/mL)	1.200 [0.300, 4.505]	1.760 [0.350, 6.415]	2.225 [0.483, 5.545]	.035
Creatine kinase‐MB on admission (IU/L)	8.33 [2.74, 26.38]	18.0 [4.45, 35.90]	17.0 [5.79, 37.35]	.004
N‐terminal pro‐brain natriuretic peptide (pg/mL)	387 [188, 1020]	474 [252, 1355]	796 [390, 2990]	<.001
Electrocardiographic findings				
ST‐segment depression ≥0.5 mm (%)	69 (28.3)	61 (47.3)	69 (63.9)	<.001
Sum of ST‐segment depressions (mm)	0.0 [0.0, 1.0]	0.0 [0.0, 3.0]	2.0 [0.0, 5.9]	<.001
Number of leads with ST‐segment depression ≥0.5 mm	0 [0, 2]	0 [0, 4]	3 [0, 5]	<.001
ST‐segment elevation in lead aVR (mm)	0.0 [0.0, 0.0]	0.0 [0.0, 0.5]	0.0 [0.0, 0.5]	<.001
Maximal QRS duration (ms)	91 [84, 99]	90 [85, 97]	93 [86, 101]	.156

*Note*: Data are presented as mean ± SD, median [interquartile range], and *N* (%).

Patients with severe LM/3VD showed a greater risk profile on admission, including advanced age, elevated heart rate, Killip class >I, and lower diastolic blood pressure, in comparison to patients who do not have LM/3VD and those without severe LM/3VD (Table 2). The laboratory findings indicated the severe LM/3VD group had elevated levels of creatine kinase MB and NT‐proBNP, in comparison to patients who do not have LM/3VD and those without severe LM/3VD (Table 2).

**TABLE 2:** Univariate and multivariate logistic regression analysis for predicting severe LM/3VD.

Variables	Odds ratio (95% CI)	*p* Value	
		Univariate	Multivariate
Age	1.050 (1.029–1.071)	<.001	<.001
Diastolic blood pressure on admission		.025	.561
Heart rate		.007	.106
Killip class >I on admission		.005	.561
Creatine kinase‐MB on admission		.049	.658
N‐terminal pro‐brain natriuretic peptide		<.001	.198
ST‐segment depression ≥0.5 mm		<.001	.239
Sum of ST‐segment depression (mm)		<.001	.941
Number of leads with ST‐segment depression ≥0.5 mm		<.001	.786
ST‐segment elevation in lead aVR (mm)	7.431 (3.862–14.301)	<.001	<.001

In addition, the ECG findings revealed that LM/3VD with high SS group exhibited a higher occurrence and a greater extent of ST‐segment depression, a greater number of leads showing ST‐segment depression, and a higher extent of ST‐segment elevation observed in lead aVR (Table 2).

Based on univariable analysis, variables that had a *p*‐value below .05 were subsequently incorporated into logistic regression analysis to identify independent predictive factors. The findings of multivariate logistic regression analysis indicated that the extent of ST‐segment elevation observed in lead aVR (OR: 7.431, 95% CI: 3.862–14.301, *p* < .001) and Age (OR: 1.050, 95% CI: 1.029–1.071, *p* < .001) were significant predictive factors of severe LM/3VD (Table 2).

### 3.2 ROC curve and AUC results

**FIGURE 1:**
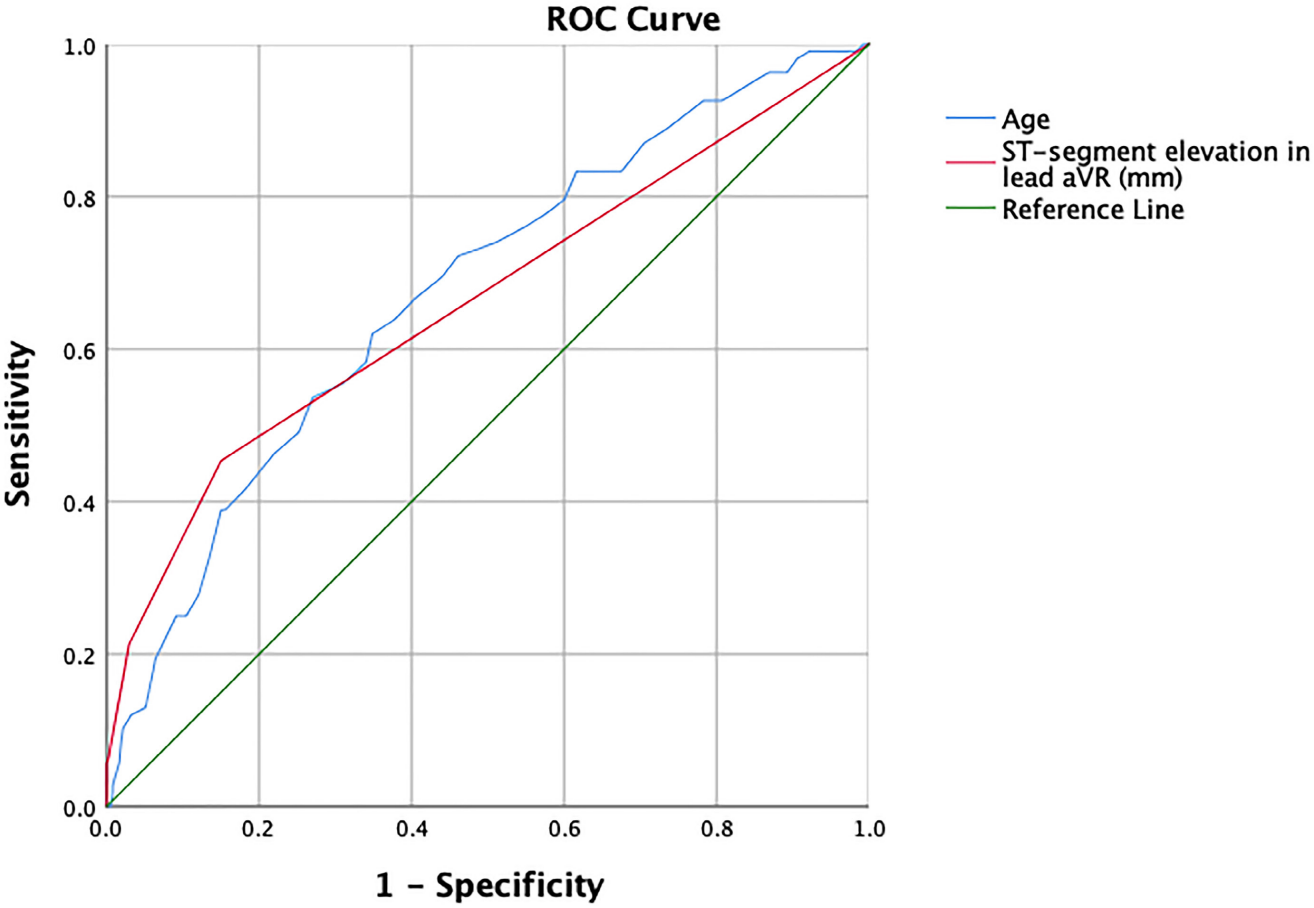
Receiver operating characteristic curves for age and degree of ST‐segment elevation in lead aVR.

**FIGURE 2:**
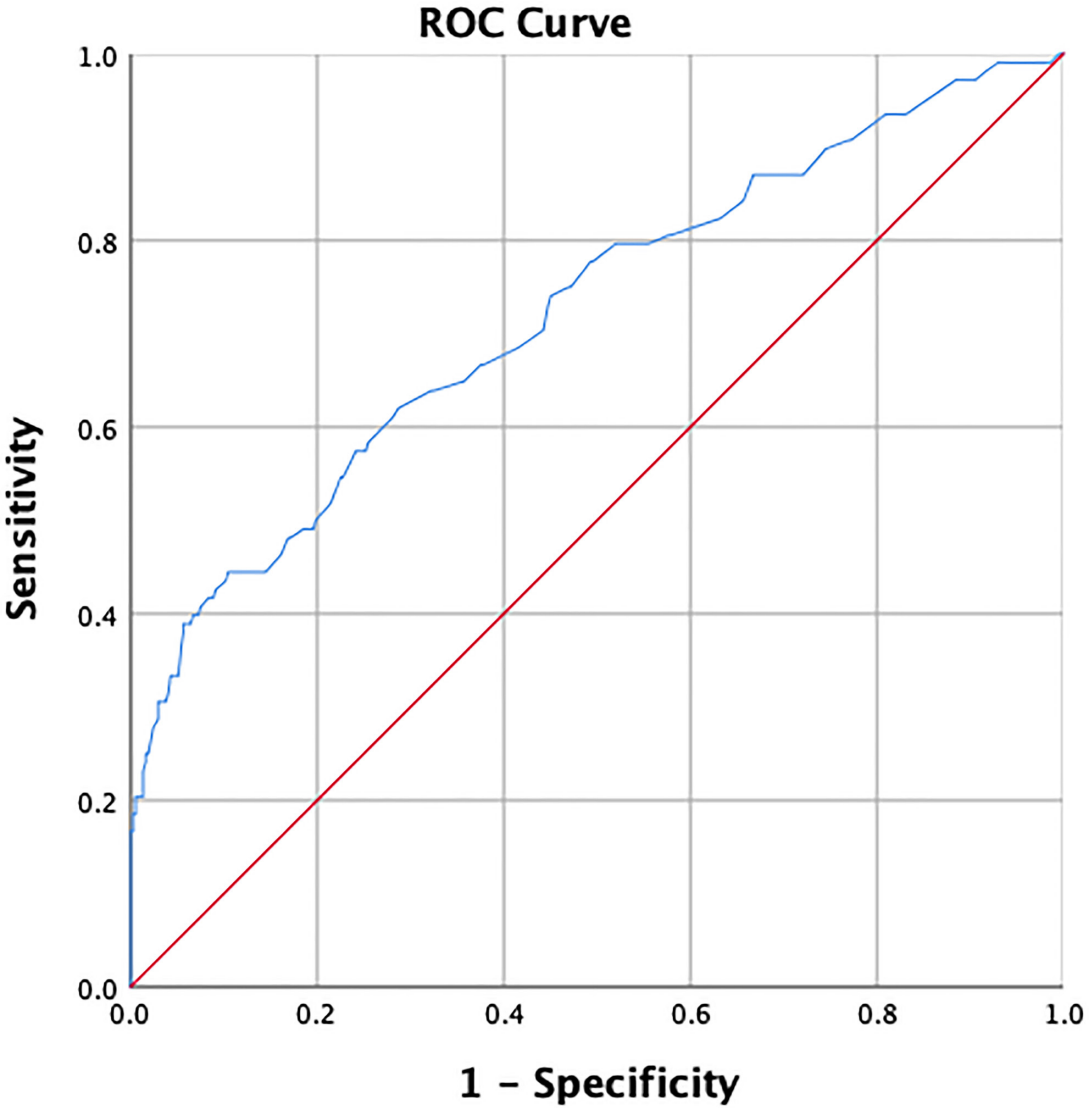
Combined (age and degree of ST‐segment elevation in lead aVR) receiver operating characteristic curve.

The optimal cut‐off points for the magnitude of ST‐segment elevation in lead aVR and age were determined to be 0.25 mm and 64.5 years, respectively. The corresponding AUC values were found to be 0.662 and 0.675, respectively (Figure 1). The combined factor of both age and the magnitude of ST‐segment elevation observed in lead aVR demonstrated a high level of discriminatory ability, as indicated by an AUC value of 0.722, suggesting strong predictive value (Figure 2).

## 4 DISCUSSION

This research aimed to assess the predictive factors associated with severe LM/3VD (SS > 22) in patients with NSTEMI who are at high risk of poor prognosis, are currently receiving a more progressive, and are more likely to be recommended for CABG. The findings of this study demonstrated that advanced age and the extent of ST‐segment elevation in lead aVR were independent predictors linked to the presence of severe LM/3VD, as defined by an SS greater than 22. Furthermore, the combination of independent predictors (extent of ST‐segment elevation in lead aVR and age) demonstrated a remarkable capacity for early prediction of severe LM/3VD, as defined by an SS greater than 22, in patients with NSTEMI.

The conventional 12‐lead ECG is a widely accessible and noninvasive clinical tool. The assessment of ECG parameters plays a crucial role in the initial evaluation of patients with acute coronary syndrome (ACS), while also providing valuable prognostic information (Burak et al., 2019; Demandt et al., 2022; Karakayali et al., 2023; Solà‐Muñoz et al., 2023; Wagner et al., 2009). Most previous research studies have primarily concentrated on evaluating the clinical significance of ST segment depression in patients diagnosed with ACS (Demandt et al., 2022; Solà‐Muñoz et al., 2023; Wagner et al., 2009). However, in patients diagnosed with NSTE‐ACS, the occurrence of ST‐segment elevation in lead aVR was indicated to have a stronger correlation with LM/3VD in comparison to ST‐segment depression in other leads (Kosuge et al., 2009, 2011; Misumida et al., 2016).

Misumida et al. (2016) revealed that ST‐segment elevation in lead aVR was found to have a significant independent association with LM/3VD in patients diagnosed with NSTEMI. Kosuge et al. evaluated the parameters of patients who were diagnosed with NSTE‐ACS. The results of the multivariate analysis indicated that the extent of ST‐segment elevation in lead aVR, the duration of the QRS complex, and the presence of positive‐troponin T were identified as predictive factors for LM/3VD (Kosuge et al., 2009). Moreover, Kosuge et al. conducted a study that revealed the significance of the degree of ST‐segment elevation in lead aVR as a predictor for the extent of LM/3VD. They also found that ST‐segment elevation in lead aVR greater than 1.0 mm strongly indicated the presence of severe LM/3VD (Kosuge et al., 2011). However, previous research did not take into account the severity of LM/3VD as assessed by SS, which has significant clinical implications for determining the most effective revascularization options regarding the use of antiplatelet medications and prognosis risk stratification. Patients with LM/3VD disease who have a low SS (≤22) have comparable treatment options with either percutaneous coronary intervention or CABG, whereas patients who have a high SS (>22) are more effectively treated with CABG (Zembala, 2019). The SS involves the comprehensive evaluation of CAD, incorporating both qualitative and quantitative assessment of angiographic parameters that consider the location and characteristics of the lesions. Several studies have provided evidence of the effectiveness of SS as a valuable tool for assessing the level of risk and predicting mortality in individuals diagnosed with ACS (Minamisawa et al., 2017; Safarian et al., 2014).

The current study explored the factors that can predict patients with severe LM/3VD, as indicated by an SS greater than 22, who are at high risk for a poor prognosis and are more recommended to require early CABG. In our study, we have provided evidence to support the association between a higher level of ST‐segment elevation in lead aVR and the presence of LM/3VD with an SS greater than 22.

ACS patients with advanced age have poorer prognosis. This predictor is additionally linked to the presence of severe coronary artery disease (Sanchez‐Nadales et al., 2023). Elderly patients exhibited a higher prevalence of severe and extensive coronary disease (Al‐Daydamony et al., 2018; D'Ascenzo et al., 2012; Salimi et al., 2023). This variable examined in our study was also identified as a predictor of LM/3VD with an SS greater than 22. This finding suggests that additional precautions should be taken for elderly patients, even among those patients who are considered to have a low‐risk profile.

To the best of our understanding, this is the first study to identify factors that can predict the early recognition of patients with severe LM/3VD, as indicated by an SS greater than 22, who have poor prognosis and are at a high likelihood of needing urgent CABG in patients diagnosed with NSTEMI. The findings of this research have significant clinical implications for early identification of patients who have poor prognosis and the selection of the most effective therapy approach in patients with NSTEMI. Patients with advanced age and ST‐segment elevation in lead aVR are associated with severe LM/3VD, as indicated by an SS greater than 22, who need to receive earlier CAG, and are more likely to be recommended for CABG.

There are several limitations in this study. First, selection bias may be present as a result of the retrospective research design. Second, the clinical characteristics included in our study were incomplete enough. Some crucial indicators, such as secondary prevention drugs, were not included in the study. Third, only patients with NSTEMI who performed coronary angiography procedures were included in this study. Fourth, the time from symptom onset to admission varied among patients. The results may have been influenced. Fifth, in our study, we did not compare the measurement data between the LM/3VD with SS ≤22 group and the LM/3VD with an SS greater than 22. Hence, further research should consider using a larger sample size and a more organized and systematic approach.

## 5 CONCLUSION

The extent of ST‐segment elevation in lead aVR and advanced age were found to be independently associated with severe LM/3VD in patients diagnosed with NSTEMI. Efforts should focus on the early identification of patients who have severe LM/3VD, in order to develop tailored aggressive management strategies.

## AUTHOR CONTRIBUTIONS

Bihan Huang, Xueying Han, Dongdong Chen, and Shaoyuan Chen provided contributions to the conception of the work. The initial draft of the document was written by Bihan Huang and Xueying Han. Yulian Huang and Dongdong Chen provided contributions to the acquisition of data for the work. The manuscript was revised and approved by Peiyi Xie and Shaoyuan Chen. The article's submission was reviewed and approved by all authors.

## FUNDING INFORMATION

This study was supported by the Natural Science Foundation of Shenzhen (grant number JCYJ20220530141815035), the Health Science and Technology Program of Nanshan District (grant number NS2022062), and the Scientific Research Projects of Huazhong University of Science and Technology Union Shenzhen Hospital (grant number YN2022025).

## CONFLICT OF INTEREST STATEMENT

No competing interest.

## 6 ETHICS STATEMENT

The study conforms to the principles of the Helsinki Declaration and has been approved by the Hospital Ethics Committee.

## Data Availability

The data that support the findings of this study are available from the corresponding author upon reasonable request.
